# Development of Social Support Networks by Patients With Depression Through Online Health Communities: Social Network Analysis

**DOI:** 10.2196/24618

**Published:** 2021-01-07

**Authors:** Yingjie Lu, Shuwen Luo, Xuan Liu

**Affiliations:** 1 School of Economics and Management Beijing University of Chemical Technology Beijing China; 2 School of Business East China University of Science and Technology Shanghai China

**Keywords:** online depression community, social support network, exponential random graph model, informational support, emotional support, mental health, depression, social network

## Abstract

**Background:**

In recent years, people with mental health problems are increasingly using online social networks to receive social support. For example, in online depression communities, patients can share their experiences, exchange valuable information, and receive emotional support to help them cope with their disease. Therefore, it is critical to understand how patients with depression develop online social support networks to exchange informational and emotional support.

**Objective:**

Our aim in this study was to investigate which user attributes have significant effects on the formation of informational and emotional support networks in online depression communities and to further examine whether there is an association between the two social networks.

**Methods:**

We used social network theory and constructed exponential random graph models to help understand the informational and emotional support networks in online depression communities. A total of 74,986 original posts were retrieved from 1077 members in an online depression community in China from April 2003 to September 2017 and the available data were extracted. An informational support network of 1077 participant nodes and 6557 arcs and an emotional support network of 1077 participant nodes and 6430 arcs were constructed to examine the endogenous (purely structural) effects and exogenous (actor-relation) effects on each support network separately, as well as the cross-network effects between the two networks.

**Results:**

We found significant effects of two important structural features, reciprocity and transitivity, on the formation of both the informational support network (*r*=3.6247, *P*<.001, and *r*=1.6232, *P*<.001, respectively) and the emotional support network (*r*=4.4111, *P*<.001, and *r*=0.0177, *P*<.001, respectively). The results also showed significant effects of some individual factors on the formation of the two networks. No significant effects of homophily were found for gender (*r*=0.0783, *P*=.20, and *r*=0.1122, *P*=.25, respectively) in the informational or emotional support networks. There was no tendency for users who had great influence (*r*=0.3253, *P*=.05) or wrote more posts (*r*=0.3896, *P*=.07) or newcomers (*r*=–0.0452, *P*=.66) to form informational support ties more easily. However, users who spent more time online (*r*=0.6680, *P*<.001) or provided more replies to other posts (*r*=0.5026, *P*<.001) were more likely to form informational support ties. Users who had a big influence (*r*=0.8325, *P*<.001), spent more time online (*r*=0.5839, *P*<.001), wrote more posts (*r*=2.4025, *P*<.001), or provided more replies to other posts (*r*=0.2259, *P*<.001) were more likely to form emotional support ties, and newcomers (*r*=–0.4224, *P*<.001) were less likely than old-timers to receive emotional support. In addition, we found that there was a significant entrainment effect (*r*=0.7834, *P*<.001) and a nonsignificant exchange effect (*r*=–0.2757, *P*=.32) between the two networks.

**Conclusions:**

This study makes several important theoretical contributions to the research on online depression communities and has important practical implications for the managers of online depression communities and the users involved in these communities.

## Introduction

### Background

Mental health problems have received more and more attention in recent years. The number of patients with mental illnesses, such as depression and anxiety disorders, is increasing rapidly worldwide [1]. A World Health Organization survey estimated that approximately 300 million people in the world might have depression by the end of 2015 [2]. How to deal with depression effectively has become a hot issue. Some studies have shown that a cost-effective way to prevent and treat depression is to obtain more social support [3]. Depressed patients with larger social support networks are more likely to improve their conditions, while patients who lack social support will gradually fall into social isolation and experience a worsening of their condition [4]. Patients often seek social support from their family, friends, and community members, but many of them keep their mental illness a secret to avoid labeling and discrimination related to depression [5].

In recent years, with the development of social networking sites, especially online health communities, patients increasingly use online resources to seek peer social support [6]. They can communicate with other patients online and develop their online social networks. On the one hand, the anonymity of online communities may make depressed patients feel more open when disclosing their illness to others [7,8]. They can more easily develop their social networks without fear of discrimination. On the other hand, peer social support in online communities is also important for patients to cope with their illness [9,10]. A survey about online depression communities showed that 41% of users thought that social support received from online communities was very helpful in treating their diseases [11].

Social support from online health communities is generally divided into informational support and emotional support [12]. Peers can provide valuable treatment information and share their own experiences to help others deal with their illness. Meanwhile, peers can show compassion and empathy to one another, which is also important for helping depressed patients to improve their symptoms [13].

Many studies have indicated that online informational and emotional support can provide huge benefits to patients with depression if they can develop these two types of social support networks. However, few researchers have studied what characteristics of patients make them more likely to develop the two types of network relationships and what patients can do to better develop these support networks. This study aimed to explore important structural features of the informational and emotional support networks and investigate which user attributes have significant effects on the formation of the two types of social network ties. The findings may help patients to better develop their social support networks to improve their conditions. In addition, although previous studies have found that both the informational support network and the emotional support network are beneficial to the improvement of mental health, few studies have examined whether there is a significant correlation between the two support networks. We wanted to find out if users could obtain more emotional support through the development of an online informational support network and vice versa. If users who are given informational support are more likely to obtain emotional support, they may be more willing to provide more information to develop their informational support network. Similarly, users may be more willing to develop an emotional support network in order to obtain more information support if the two supports align with one another. Therefore, another aim of this study was to examine whether there is an association between the informational support network and the emotional support network. The findings may help patients to better understand the relationship between the two support networks, which may help them better develop their social support networks to improve their conditions.

Over the years, scholars have used some of the quantitative methods developed for social network analysis to better understand social networks in online communities. In particular, the use of exponential random graph models (ERGMs) is quickly becoming recognized as one of the central approaches in analyzing social networks [14]. ERGMs are tie-based models for understanding how and why social network ties arise. ERGMs can incorporate different types of network configurations and estimate their effects on network formation. For example, ERGMs can incorporate any number of binary, categorical, and continuous actor attributes to determine whether actor attributes are associated with the formation of network ties. As well, we could extend ERGMs to multivariate analysis of two networks and examine the cross-network effects [15]. ERGMs are concerned first and foremost with explaining the patterns of ties in a social network and thus provide a framework within which hypotheses about the impact of various factors on social tie formation can be statistically examined. Therefore, in this study, we applied ERGMs to social support networks in online depression communities in our attempt to investigate the following research questions (RQs):

- RQ1: What are important structural features of the informational support network and the emotional support network in online depression communities? Which user attributes will affect the formation of the two types of social ties?
- RQ2: Is there an association between the two social support networks?

### Theoretical Background and Research Hypotheses

ERGMs are tie-based models for understanding how and why social network ties arise [14]. The models are based on some theoretical assumptions about social networks: network ties not only self-organize, but also are influenced by actor attributes and other exogenous factors. Therefore, in this study, we wanted to examine the formation of social network ties in online depression communities from the following aspects: network self-organization, individual attributes, and exogenous contextual factors.

#### Network Self-Organization

One particularly important feature of social networks is that network ties depend on one another, which is referred to as network self-organization. That is to say, the presence of one tie may affect the presence of other ties. We need to take account of purely structural tendencies for tie formation in the contexts of online depression communities. Two common parameters for purely structural effects were included in our study: reciprocity and transitivity.

The reciprocity principle refers to the phenomenon that people like those who like them [16]. Reciprocity is seen as a basic and universal human behavior, and social ties are generally expected to be reciprocated in human social networks [17]. We thought that the reciprocity effect could occur in the context of online depression communities. On one hand, we expect social ties to be reciprocated in the informational support network. When patients communicate with peers with the same illness to share valuable information and experiences, they will expect the mutual reciprocity that justifies their expense in terms of time and effort spent contributing their knowledge. The users who receive valuable information from their peers are more likely to reciprocate the information providers with their knowledge. On the other hand, we expect social ties to be reciprocated in the emotional support network. When patients receive blessings and encouragement from other members, they will thank their peers and give back the blessings they receive. They will develop reciprocated ties to encourage each other to fight against the illness together. Based on the arguments above, we proposed the following hypotheses:

- Hypothesis 1a: Patients in online health communities tend to provide informational support to each other based on the principle of reciprocity.
- Hypothesis 1b: Patients in online health communities tend to provide emotional support to each other based on the principle of reciprocity.

Transitivity is another important feature of most social networks and describes the tendency in a social network for the friend of a friend to become one’s friend [18]. In the context of online depression communities, when one user makes a post to present information, share ideas, or express emotions, other members will follow the post to engage in the discussions or exchanges. Therefore, it is very possible that the members participating in the discussion of the same topic will develop close friend relationships. Based on the arguments above, we proposed the following hypotheses:

- Hypothesis 2a: Patients are more likely to form new informational support ties with those who share mutual friends based on the principle of transitivity.
- Hypothesis 2b: Patients are more likely to form new emotional support ties with those who share mutual friends based on the principle of transitivity.

#### Individual Attributes

Individual attributes in social network theory play very important roles in the formation of social ties [19-21]. Studies have shown that some common demographics, such as gender, age, education, and income levels, affect the involvement of individuals in social activities [22]. In addition, some other individual factors such as motivations and attitudes toward others in their social networks also have an impact on their social tie formation. We use the term actor attribute effects to explore the association of some specific individual attributes with social ties.

The homophily principle states that people are more likely to form social ties with others who share similar characteristics [23]. The literature on the phenomenon comes from social network studies that were conducted in some specific research fields. In this study, we proposed gender to be the most influential source of homophily. First, gender is the most extensively researched factor among demographic characteristics of patients with depression. In recent years, the literature on depression has reflected great interest in gender differences not only in depression symptoms but also in the perceived support [24-26]. It was found that there are significant gender differences both in the quality of perceived support and in the importance of support variables as predictors of depressive symptoms [27-29]. Second, users in online social networks often consider gender as an important factor in their interpersonal communication with other community members [30]. By contrast, most of the other personal demographic information (eg, age, race, and occupation) is not considered to be of great help in enhancing communication between users. Therefore, users will probably fill in their gender but leave the other demographic options unmarked when registering on the website because they are unwilling to risk their personal privacy. Therefore, it is perhaps reasonable that we only consider gender as the source of the homophily in this study.

According to the existing theories research on gender differences in depression, we proposed that gender differences in psychological factors, such as coping style (emotion-focused coping or problem-focused coping), could play an important role in influencing patients’ motivation and behavior in online depression communities. In addition, it is widely recognized that there are significant gender differences in the perception and utilization of social support [31,32]. We have reasons to believe that informational support and emotional support from online communities may have different effects on male and female patients and members of the same gender are more likely to provide social support to one another. Based on the arguments above, we proposed the following hypotheses:

- Hypothesis 3a: Patients of the same gender are more likely to form informational support ties.
- Hypothesis 3b: Patients of the same gender are more likely to form emotional support ties.

Social influence plays an important role in the formation of social networks. There have to be some influential people in social networks who have a disproportionate influence on others. Those influential users in online social networks could be identified based on centrality measures according to social network theory [33]. Users with high degree centrality scores can be characterized as being highly informed or well-connected individuals [34]. Social capital theory suggests that influential users might have more opportunities to influence the behavior of other users to accumulate social capital, such as being respected by other users. We have reasons to believe that in online depression communities, users who have great influence are more likely to get more social capital, including informational support and emotional support. In addition, the more that users are embedded in social networks, the more easily they will get social support. Online time is an important indicator of the degree of embedding in social networking networks. Users with more online time have more opportunities to participate in interactive activities on online platforms. They are well known by the members of the community, so it is easier for them to get social support. Based on the arguments above, we proposed the following hypotheses:

- Hypothesis 4a: Users who have great influence are more likely to form informational support ties.
- Hypothesis 4b: Users who have great influence are more likely to form emotional support ties.
- Hypothesis 5a: Users with more online time are more likely to form informational support ties.
- Hypothesis 5b: Users with more online time are more likely to form emotional support ties.

Users’ social activities will have an important impact on the formation of their online social networks. According to the preferential attachment model—a widely accepted mechanism that accounts for tie formation in social networks—actors with a large number of existing ties are more likely to attract connections from other actors joining the network, thus showing the phenomenon that “the rich get richer” [35,36]. In online depression communities, users develop social relationships by participating in social activities including making and replying to others’ posts. Those users with high online activity levels generate a lot of posts and are considered to be core nodes in the social network, so they have more chances of getting more social support. A study based on online weight loss networks showed that active users received a high level of both informational and emotional support [37]. Based on the arguments above, we proposed the following hypotheses:

- Hypothesis 6a: Users who write more posts are more likely to form informational support ties.
- Hypothesis 6b: Users who write more posts are more likely to form emotional support ties.
- Hypothesis 7a: Users who provide more replies to other posts are more likely to form informational support ties.
- Hypothesis 7b: Users who provide more replies to other posts are more likely to form emotional support ties.

In addition, some studies pointed out that newcomers who recently joined online health communities get more attention easily [38]. On the one hand, it is generally thought that newcomers lack the necessary knowledge, and thus they are more anxious to seek help and the emotional support of community members [39]. On the other hand, we believe that the experienced “old-timers” are more willing to share knowledge with newcomers and provide them with more support for a number of reasons [40-42]. First, some of them hope to develop new social ties with newcomers to enhance their social capital [43]. Second, community members with a high degree of shared identity and strong levels of trust consider it a duty to assist newcomers in improving depression treatment and outcomes and they hope that the newcomers soon became fully integrated into the online community [44,45]. Third, community members are more willing to share information and provide emotional support based on an altruistic motivation to provide help to newcomers who badly need it because altruism is more likely to occur when the recipient is in greater need or is more likely to profit from an altruistic act [46,47]. Based on the arguments above, we proposed the following hypotheses:

- Hypothesis 8a: Newcomers are more likely to form informational support ties.
- Hypothesis 8b: Newcomers are more likely to form emotional support ties.

#### Exogenous Contextual Factors

Some exogenous contextual factors may be important to tie formation. We often treat these as tie covariates [48]. For example, when there are multiple network ties, different types of networks may interact with each other and these interactions will affect the structure of each network. In this case, a certain social network, as an exogenous contextual factor, may be considered a tie covariate of another social network. In the context of online depression communities, there are mainly two social support networks: informational support networks and emotional support networks. We sought to determine whether there is an association between the two networks and if they are tie covariates of each other. That is, emotional support ties may be affected by the presence of informational support relations and vice versa.

The most fundamental cross-network effects for directed networks are entrainment and exchange effects [49]. On one hand, we want to examine whether the two social support networks are entrained, so that users who obtain informational support are more likely to obtain emotional support. It is possible that many patients need both informational support and emotional support when they ask for help or, alternatively, that support providers are more inclined to provide emotional support to those network partners who need informational support. On the other hand, the two support networks may be exchanged, in which case those who receive informational support tend to give emotional support to those informational support providers. It is also reasonable for users to develop reciprocal relationships, and they will express appreciation and provide emotional support to those peers who have helped them. Based on the arguments above, we proposed the following hypotheses:

- Hypothesis 9: The two social support networks may be entrained so that users who obtain informational support are more likely to obtain emotional support and vice versa.
- Hypothesis 10: The two social support networks may be exchanged so that users who receive informational support are more likely to give emotional support to those informational support providers and vice versa.

## Methods

### Research Context and Data Collection

One of the most popular online health platforms for Chinese patients with depression was chosen as the data source. The online discussion forum was comprised of 15 discussion boards where patients could talk about a variety of topics related to depression. After over 10 years of development, the platform has attracted more than 10,000 registered users. The website is an open information exchange platform for patients with depression, where they can discuss their symptoms of depression, share their own treatment experiences, and seek useful medical information. In addition, the website provides an emotional communication channel for patients with depression, in which they can open their hearts to express their feelings and thoughts with their peers, such as expressing their emotional distress, showing sympathy, and encouraging one another. Therefore, the informational and emotional support networks developed in the online depression community have played a very important role in improving the condition of patients with depression.

We used the Java WebCrawler script to collect the webpage information from the online depression community and then parsed the webpages to obtain available information. We obtained a total of 74,986 original posts and replies to the posts created by the users in the online community from April 2003 to September 2017. The available information about the posts was stored into a database, including the author’s ID, the post’s title and body content, and the time stamp. In addition, some user profile information available to the public was also stored, such as gender, online points, online duration, number of posts and replies written by the user, and registration time. It should be noted that some ambiguous or incomplete posts were present on the discussion boards. For example, some nonbinary individuals or patients who did not disclose their gender left the gender option unmarked. A total of 9452 ambiguous or incomplete posts were identified and excluded from the experimental data.

Considering the potential risk to privacy and confidentiality, we only used the information that was available to the general public. No user identification data, such as names and ID numbers, was used to ensure that there was no risk of sensitive information disclosure. Therefore, our study had minimal risk to human subjects and followed core ethical principles. In addition, we took some measures to make sure that the users involved were fully informed about our study. First, an official notification elaborating on the research and how the user information would be used was sent by an internal email to users to confirm that it had been read. Second, to fulfill the ethical requirements, one page with a “click to accept” button was sent to the users through the messaging system on the platform, which allowed them to click the button to express their agreement to participate in our research.

Some key variables related to user attributes were measured in our empirical analysis. The variable of “gender” was measured as a dummy variable, with 1=male and 0=female. We used the variable of “influence” to represent the online influence of the users, which was measured in terms of online points, indicating the contribution made by the member to the website. Some other variables that were used in our empirical analysis—online duration, and number of posts and replies of users—were measured in terms of time spent on the website, the number of original posts made by the user, and the number of replies to other posts written by the user. In addition, the variable of “newcomers” was measured as a dummy variable, with 1=users who were among the most recent 25% of individuals to join the website and 0=others.

**Figure 1:**
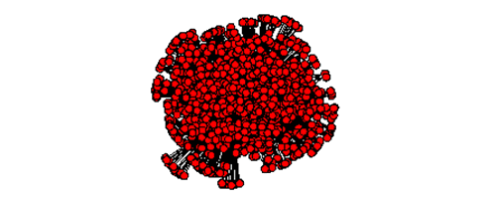
Informational support network.

**Figure 2:**
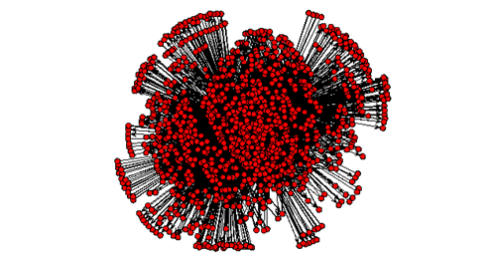
Emotional support network.

We further performed a content analysis of all the original posts and classified them into informational and emotional posts. We extracted some keywords related to the diagnosis and treatment of depression to construct an information dictionary and then used the dictionary-based method to distinguish informational posts. In the same way, we extracted some keywords indicating emotional support to construct an emotion dictionary and then used the dictionary-based method to distinguish emotional posts. For the list of keywords in the information and emotion dictionaries, see Multimedia Appendix 1. After controversial posts were deleted from the experimental data, an informational support network of 1077 nodes and 6557 arcs and an emotional support network of 1077 nodes and 6430 arcs were constructed (Figures 1 and 2, respectively). We can see that the emotional support network has a stronger core-periphery structure than the informational support network, indicating that users preferred to exchange information with other users in the forum, while they were more likely to derive emotional support from the broader population.

### ERGMs

ERGMs are a class of statistical models for social networks that account for the presence or absence of network ties [50-52]. ERGMs are particularly useful for overcoming the limitations of traditional regression methods, which are ill-suited for analyzing network data because ERGMs do not require the assumption of independence among the ties in a network. In addition, EGRMs have many advantages in social network analysis [53,54]. First, ERGMs can incorporate different types of local patterns of ties, which are also called “network configurations,” and estimate the effects of these network configurations on the formation of network ties. Second, ERGMs can accommodate any number of binary, categorical, or continuous actor attribute variable and dyad-specific covariates and determine whether they are associated with the formation of network ties. Third, ERGMs can also be used to analyze different types of networks with various types of nodes and relationships and can even model the two networks simultaneously. Therefore, ERGMs are novel and powerful tools for analyzing and explaining the patterns of network ties, especially in complex social networks [54].

We used the notation and terminology described by Robins [14]. For each pair (i and j) of a set number of actors, X_ij_ is a random variable that represents a tie between actor “i” and actor “j” (X_ij_=1 if there is a tie between actors i and j, and 0=no tie). These ties are represented in an n×n adjacency matrix (with n being the number of actors in the network), which is denoted as X. We specify x_ij_ as the observed value of X_ij_, and x denotes a matrix of observed ties in the network. ERGMs have the following general form:


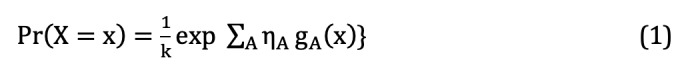


The A refers to a certain type of network configuration and is composed of a set of nodes and ties among them. The g_A_(x) represents network statistics corresponding to configuration A. For the ERGM used in this study, g_A_(x) is the number of configurations A observed in the network. The η_A_ coefficient is the parameter to be estimated corresponding to configuration A. The k is a normalizing constant to ensure a proper probability distribution.

**Figure 3:**
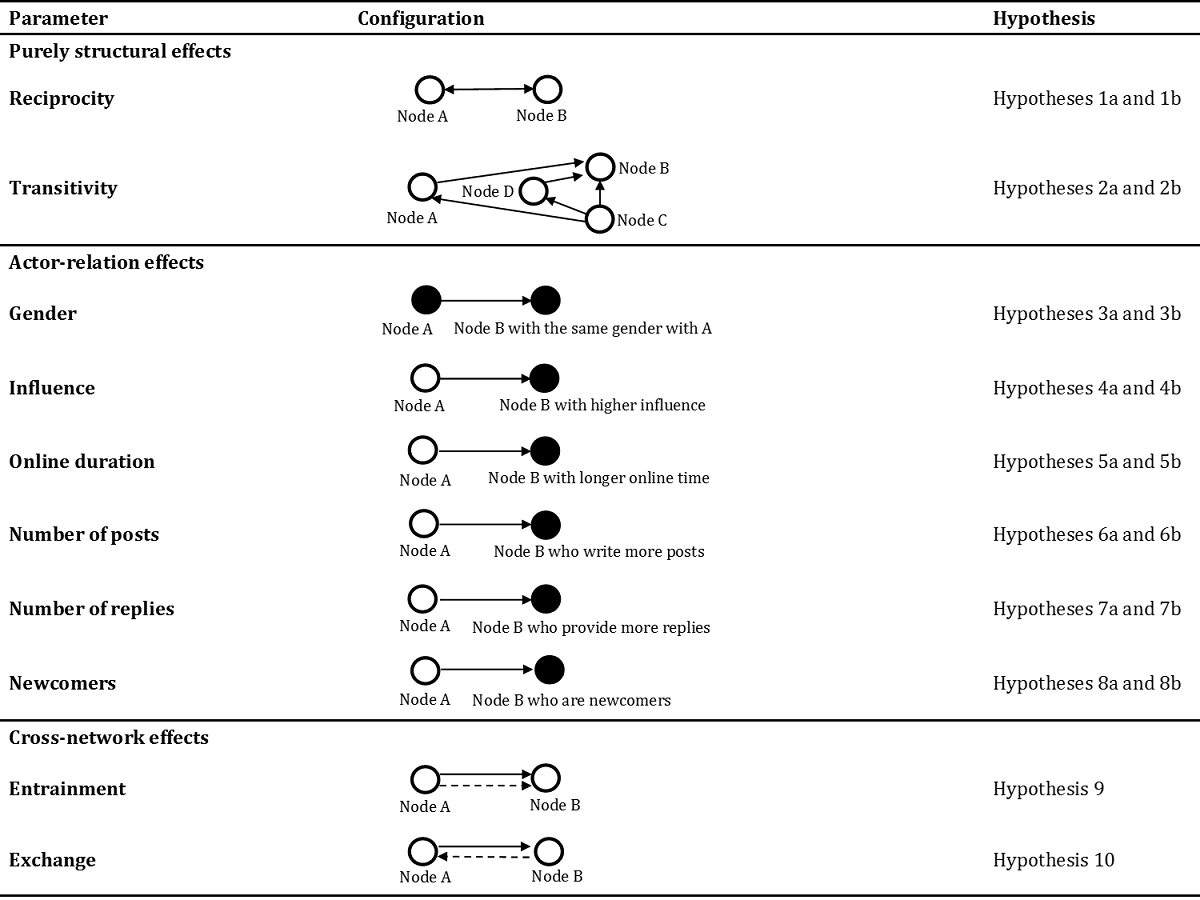
Summary of network configurations included in the exponential random graph model.

To better understand how to use ERGMs to test our hypothesis, we provide a graphical presentation of purely structural effects, actor-relation effects, and cross-network effects used in the model along with the corresponding research hypotheses (Figure 3).

## Results

We estimated the ERGMs using Markov chain Monte Carlo maximum likelihood estimation (MCMC-MLE) methods and implemented the simulation-based algorithms for MCMC-MLE in the statnet software suite developed for the R platform. The estimation procedure successfully converged for all parameters presented for the two social support network models.

Table 1 presents the results of ERGM estimates for the informational support network, which shows the parameters for which there was a significant effect (ie, when the parameter estimate was greater than two times the standard error in absolute value). The results show that the estimates for the purely structural effects of reciprocity and transitivity were significant for the informational support network, indicating that patients in the online depression community tended to provide informational support to each other and were more likely to form new informational support ties if they shared mutual friends. Thus, hypotheses 1a and 2a were supported.

**Table 1:** Exponential random graph model estimates for the informational support network.

Parameter		Estimate	SE	*P* value	Hypothesis	Result
**Purely structural effects**						
	Reciprocity	3.6247^a^	0.1937	<.001	Hypothesis 1a	Supported
	Transitivity	1.6232^a^	0.0102	<.001	Hypothesis 2a	Supported
**Actor-relation effects**						
	Gender	0.0783	0.0617	.20	Hypothesis 3a	Not supported
	Influence	0.3253	0.1681	.05	Hypothesis 4a	Not supported
	Online duration	0.6680^a^	0.1562	<.001	Hypothesis 5a	Supported
	Number of posts	0.3896	0.2132	.07	Hypothesis 6a	Not supported
	Number of replies	0.5026^a^	0.1505	<.001	Hypothesis 7a	Supported
	Newcomers	–0.0452	0.1036	.66	Hypothesis 8a	Not supported

^a^Significant effect.

We then interpreted the ERGM results for the actor-relation effects in the informational support network. We found that there was no significant homophily effect for gender, indicating that there was no empirical evidence that patients of the same gender were more likely to form informational support ties. Thus, hypothesis 3a was not supported. In addition, the results showed that no significant effects for influence, number of posts, or newcomers were obtained, indicating that there was no tendency for users who had great influence or wrote more posts or were newcomers to form informational support ties more easily. Thus, hypotheses 4a, 6a, and 8a were not supported. However, we found significant effects for online duration and number of replies. This suggests that users who spend more time online or those who provide more replies to other posts are more likely to form informational support ties. Thus, hypotheses 5a and 7a were supported.

Table 2 presents the results of ERGM estimates for the emotional support network. The results for purely structural effects show that the parameter estimates for reciprocity and transitivity were significant for the emotional support network, indicating that patients in online depression communities tend to provide emotional support to each other and are more likely to form new emotional support ties if they share mutual friends. Thus, hypotheses 1b and 2b were supported.

**Table 2:** Exponential random graph model estimates for the emotional support network.

Parameter		Estimate		SE		*P* value		Hypothesis		Result	
**Purely structural effects**											
	Reciprocity		4.4111^a^		0.2991		<.001		Hypothesis 1b		Supported
	Transitivity		0.0177^a^		0.0008		<.001		Hypothesis 2b		Supported
**Actor-relation effects**											
	Gender		0.1122		0.968		.25		Hypothesis 3b		Not supported
	Influence		0.8325^a^		0.1229		<.001		Hypothesis 4b		Supported
	Online duration		0.5839^a^		0.1333		<.001		Hypothesis 5b		Supported
	Number of posts		2.4025^a^		0.2147		<.001		Hypothesis 6b		Supported
	Number of replies		0.2259^a^		0.1113		.04		Hypothesis 7b		Supported
	Newcomers		–0.4224^a^		0.1165		<.001		Hypothesis 8b		Not supported

^a^Significant effect.

We then interpreted the ERGM results for the actor-relation effects in the informational support network. We found that there was no significant homophily effect for gender, indicating that there was no empirical evidence that patients of the same gender are more likely to form emotional support ties. Thus, hypothesis 3b was not supported. For other attribute-related effects, significant positive estimates were obtained for the following four parameters: influence, online duration, number of posts, and number of replies. This suggests that users who have great influence, spend much time online, write more posts, and provide more replies to other posts are more likely to form emotional support ties. Thus, hypotheses 4b, 5b, 6b, and 7b were supported. However, we found significant and negative effects for newcomers, indicating that newcomers are less likely than experienced old-timers to receive emotional support. Thus, hypothesis 8b was not supported.

We then used a bivariate ERGM to model the two social support networks simultaneously and performed the simultaneous analysis of the multirelational network structure using the XPNet program, a multirelational version of the PNet program. Table 3 presents the results of ERGM estimates of cross-network effects.

**Table 3:** Exponential random graph model estimates of cross-network effects for the two social support networks.

Parameter	Estimate	SE	t-ratio	Hypothesis	Result
Entrainment	0.7834^a^	0.2422	427.403	Hypothesis 9	Supported
Exchange	–0.2757	0.3219	367.969	Hypothesis 10	Not supported

^a^Significant effect.

Our motivation for studying these two networks simultaneously was to investigate whether the informational support network aligns with the emotional support network in the context of online depression communities. In particular, we sought to examine if the two networks are entrained or exchanged, since entrainment and exchange are the two key bivariate effects of directed networks.

As seen in Table 3, the multivariate network effects revealed that the two networks are likely to be entrained, which can be seen from the positive and significant entrainment effect. This suggests that users who obtain informational support are more likely to obtain emotional support, and vice versa. Thus, hypothesis 9 was supported. However, the two relations were not exchanged, which was demonstrated by a nonsignificant parameter estimate, indicating that there was no significant evidence that users who received informational support were more likely to give emotional support to those informational support providers and vice versa. Thus, hypothesis 10 was not supported.

**Figure 4:**
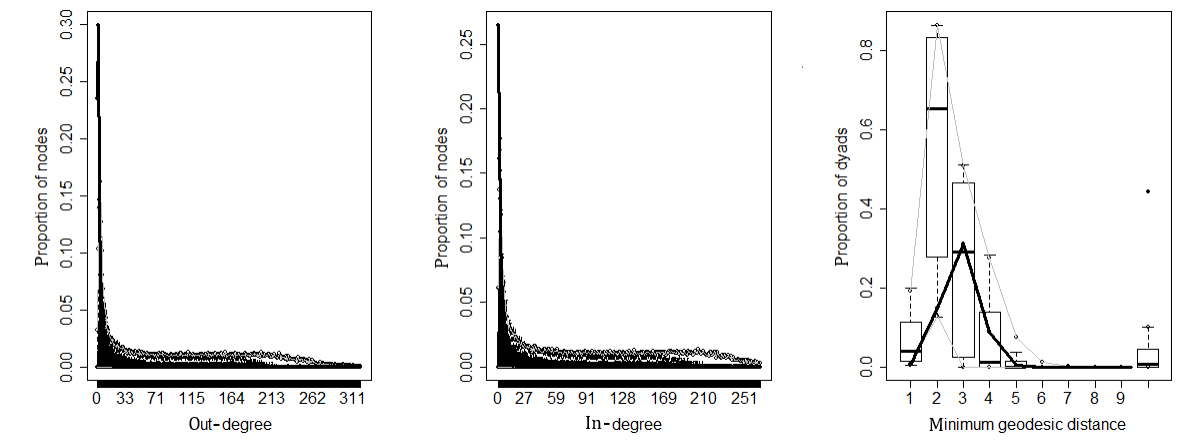
Goodness of fit for measures from simulated informational support networks.

**Figure 5:**
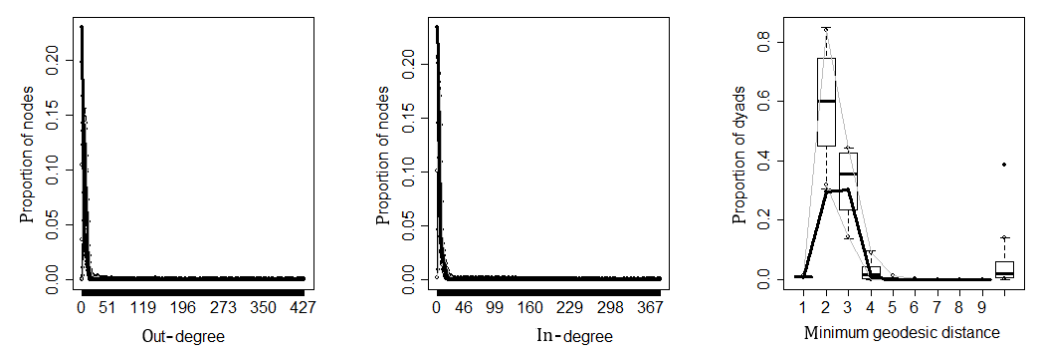
Goodness of fit for measures from simulated emotional support networks.

To examine whether the ERGMs in the study fit the observed network well, we employed graphical evaluations of the goodness of fit to visualize the match between the predicted and observed networks (Figures 4 and 5). In the plots, the thick black line represents the observed network and the gray lines show the 95% confidence interval of simulated network measures. When the black line falls between the gray lines, the simulated networks are capturing the characteristics of the observed network. The first and second plots in Figures 4 and 5 represent the out-degree distribution and in-degree distribution, respectively. The goodness of fit for out-degree and in-degree showed that observed and simulated networks were not significantly different. The third plot displays another network statistic, the distribution of geodesic distances, which represents the pairwise shortest distances between nodes, and also illustrates that the models provide a good fit between the simulated and observed networks.

## Discussion

### Principal Findings

This article examined endogenous effects (purely structural effects) and exogenous effects (actor-relation effects) on the formation of the informational and emotional support networks of patients in online depression communities separately and then explored cross-network effects between the two networks. Some valuable findings were obtained as follows.

First, the results of this study provide support for the effects of some important structural features on the formation of two support networks. We found that social ties in online depression communities are reciprocal and transitive, which is in line with the findings of network theories about tie formation showing that reciprocity and transitivity are seen as the basic principles of social interaction [18]. Reciprocation and transitive closure lead us to suggest that users in online depression communities prefer to develop social ties through information interaction and mutual emotional encouragement and are also willing to form new social relationships with users who share mutual friends. The result is in accordance with the previous studies [55-57], proposing that these structural effects will help the diffusion of information and create an atmosphere of mutual support, as well as promote the sustainable development of online depression communities.

Second, we examined the effects of some individual factors on the formation of the two networks and found that actor-relation effects may differ between the two networks. Gender is not an important determinant of the formation of either the informational support network or the emotional support network. Users were willing to communicate not only with people of the same gender but also with the opposite sex. The results are in accordance with some previous research about gender-typical behaviors and cross-gender friendships. Some studies pointed out that men consider their cross-gender friendships as expressive, whereas women consider their cross-gender friendships as, if anything, instrumental [58]. Thus, we have reason to believe that male users are also likely to develop emotional support networks with female users, not just with male users, while female users are also likely to develop informational support networks with male users, not only with female users. On one hand, women usually have a stronger sense of community identification and feel more responsible than men toward other members [59]. Men tend to be more open, more self-disclosing, and more intimate with female friends than with male friends [58]. Therefore, it is possible that male users in online depression communities may receive more emotional support from female users. For example, one study found that men living without a spouse in the household were more likely to lack emotional support and were more vulnerable to depression than women in the same situation [28], and thus men living without a spouse are more likely to develop an emotional network with female users. On the other hand, according to sex role theory, which posits that the female gender role is associated with less power and lower social status [60,61], women are more likely to need informational support. Compared with women tending to use strategies that modify their emotional response, men tend to deal with depression by problem-focused coping [62], so it is possible that female users in online depression communities may receive more informational support from male users. The results are in line with the earlier finding that male users’ postings are usually more professional and contained more professional knowledge than female users’ postings in online health communities [30], so both male and female users are more inclined to develop informational support networks with male users.

For other attribute-related effects, we found that users who spent a lot of time online were more likely to form both informational and emotional support ties. A possible explanation is that users with more online time are considered to be centrally embedded in the network. Scholars have pointed out that a person’s position in the network influences his/her willingness and ability to communicate with others [63]. The closer a person is to the center of the network, the more he/she has opportunities to participate in interactive activities to provide informational and emotional support with other members. We also found that those users who provide more replies to other posts are more likely to form both informational and emotional support ties. A possible explanation is that users can accumulate their own social capital by actively interacting with others. According to social capital theory, users would like to provide more replies to other posts when they perceive that doing so enhances their online reputations [64]. Accumulated reputation could bring them certain indirect benefits, such as becoming known to community members, thereby potentially increasing their opportunities to obtain informational and emotional support. However, some individual attributes have different effects on the formation of different networks. We discovered evidence that users who had great influence and wrote more posts were more likely to develop emotional support ties, but there was no tendency for these users to develop informational support ties more easily. A possible explanation is that these users were patients who had experienced long-term struggles with depression and thus had first-hand experience of preventing and dealing with the disease. These users with a high level of expertise preferred to contribute their knowledge and experience to help others rather than to obtain information. However, these contributors were likely to develop stronger emotional ties than others. According to the norm of reciprocity in social capital theory, contributors expect the mutual reciprocity that justifies their expense in terms of time and effort spent contributing their own knowledge [65]. Therefore, it is reasonable to believe that they are likely to receive more emotional support because their contribution efforts will be reciprocated by other members.

In addition, we found that newcomers did not get more informational support than old-timers and they were even less likely than old-timers to receive emotional support. It is possible that newcomers lack the necessary skills to make use of social networks to obtain information. In the meantime, they receive less attention because of the lack of accumulation of social capital, making it difficult for them to get more emotional support. This argument is supported by a recent study by Lu et al [43], which found that it is difficult for newcomers involved in online depression communities to increase their social capital in a very short period of time and they will take a considerable amount of time contributing to the online community to establish mutual trust with other members.

Finally, we examined the association between the two social networks and the results revealed that the informational support network does not always align with the emotional support network in the context of online depression communities. There was a significant entrainment effect and a nonsignificant exchange effect between the two networks. This suggests that users who obtain informational support are more likely to obtain emotional support simultaneously. The result is in line with the discussion above, suggesting that users can accumulate their own social capital through long-term participation in online communities or actively interacting with others, and those with more social capital will more easily receive informational and emotional support simultaneously [64]. However, the reverse statement is not necessarily true. That is to say, users don’t necessarily provide informational support to those who provide them with emotional support. A possible explanation is that for users with lower social status and newcomers, they lack the necessary knowledge and it is difficult for them to provide effective informational support to others [66-69]. When they obtain emotional support from members, they are more inclined to develop reciprocal relationships and also to provide emotional support to those who provide them with emotional support, such as encouraging each other to fight against the illness together.

### Limitations

This study has some limitations. First, the ERGMs used in this study were cross-sectional models, but more information about dynamic social processes could not be obtained from a cross-sectional view. We should consider extending the ERGMs to longitudinal data in further studies. Second, we mainly focused on the informational support network and the emotional support network. However, users in online depression communities may establish all kinds of social ties. For example, adding friends and following celebrities will probably form social ties. It is worth further study how these social ties will affect the formation of the two support networks. Third, we consider that other demographic factors, including age and location of residence, may also be important sources of homophily. However, considering that it was difficult to obtain empirical data on demographics in our sample, it is temporarily unfeasible to study the other demographic factors. We will consider the issue in further studies. Finally, we only used a binary classification for gender in the study. While there are clear pragmatic reasons for this, it is well known that individuals with sexual or gender orientations that are seen to deviate from the norm may experience higher rates of depression. This should be taken into account in future research.

### Conclusions

The online depression community is increasingly seen as a promising communication platform for patients suffering from depression, where they can exchange valuable medical information to form an informational support network and provide emotional support to one another to form an emotional support network. While many studies focus on the benefits of social support networks, however, little is known about how patients with depression develop social support networks through online health communities. This paper attempted to apply social network theories to examine which endogenous and exogenous factors will affect the formation of the two support networks and whether there is an association between the two networks. ERGMs were used in our study to test the hypotheses about the effects of network structure and individual attributes on the formation of the informational and emotional support networks. We then chose a popular online health platform for Chinese patients with depression as the data source to empirically test the proposed hypotheses. The results showed some important effects of structural features—namely reciprocity and transitivity—on the formation of the support networks. The results also provided support for the effects of some individual factors on the formation of the two networks respectively. For example, users who spent a lot of time online and provided more replies to other posts were more likely to form both informational and emotional support ties. However, some actor-relation effects may differ between the two networks. For example, we discovered evidence that the users who had great influence and wrote more posts were more likely to develop emotional support ties, while there was no tendency for these users to develop informational support ties more easily. In addition, we examined the association between the two social networks and found that the informational support network did not always align with the emotional support network in the context of online depression communities. There was a significant entrainment effect and a nonsignificant exchange effect between the two networks.

This study makes several important theoretical contributions to the research on online depression communities. First, our research provides new insights into online social support from online depression communities. Recent studies have suggested that online social support can bring considerable benefits to patients with depression [9-11]. However, few studies have focused on the mechanism of the formation of social relationships or explored which factors have significant effects on the formation of social support ties. This study aimed to investigate what types of patients are more likely to form social ties in online depression communities and to help them develop online social ties to improve their conditions. Second, our research contributes to the previous research by adopting social network theories to analyze the social support networks in the context of online depression communities. In this study, we divided the social support networks into the informational support network and emotional support network, and proposed that network self-organization, individual attributes, and exogenous contextual factors have significant effects on the formation of the two social support networks. Third, we developed a new theoretical model by applying ERGMs to analyze important structural features and user attributes that affect the formation of social networks. Eighteen research hypotheses were proposed and empirically tested. The empirical results reveal that some of the hypotheses were supported whereas others were not. The findings help us to better understand the formation of the two social networks.

Our research has important practical implications for the managers of online depression communities and the users involved in these online communities. First, the empirical results help the managers to better understand online social networks and take specific measures to develop social support networks in online depression communities. The results revealed that social ties in online depression communities are reciprocal and patients prefer to communicate and share experiences with peers who have the same conditions. Thus, the managers may provide humanized supporting functions to facilitate community members to find their peers who have the same conditions. The results also revealed that social ties are transitive and patients are more likely to form new informational support ties with individuals who share mutual friends. Thus, the managers may provide them with more opportunities to discover topics that interest them and create a welcoming atmosphere in which patients can easily share ideas and express emotions with each other. Second, our results make it clear what patients should do to better develop the two types of support networks. We found that long-term users and those who provide more replies to other posts are more likely to form informational support ties, while those users who had great influence, spend much time online, write more posts, and provide more replies to other posts are more likely to form emotional support ties. These findings may help patients to better develop their social support networks to improve their conditions.

## Appendix

Multimedia Appendix 1 Keywords in the information dictionary and emotion dictionary.

## Abbreviations

ERGM: exponential random graph model
MCMC-MLE: Markov chain Monte Carlo maximum likelihood estimation
RQ: research question
