# Supplementary material for: Development of Social Support Networks by Patients With Depression Through Online Health Communities: Social Network Analysis
Source: JMIR Med Inform. 2021 Jan 7;9(1):e24618. doi: 10.2196/24618 (PMC7819780; doi:10.2196/24618)
Supplement: Multimedia Appendix 1 [file medinform_v9i1e24618_app1.docx]

Appendix 1. Keywords in the information dictionary and emotion dictionary

| Social support | Keywords |
| --- | --- |
| Informational support | treatment, side effect, counseling, medicine, Fluoxetine Hydrochloride Pulvules, MirtazapineTablets, Tianeptine, Trazodone Hydrochloride Tablets, Lexapro, Venlafaxine, Setraline, Duloxetine, Lithinm Carbonate, Clonazepam, Paroxetine Hydrochloride, Quetiapine fumarate, Morita Therapy, Cognitive Therapy, Hypnotherapy, Block Therapy, Social phobia, Bipolar disorder, schizophrenia, obsessive compulsive disorder(OCD), doctor, medical treatment, Bipolar disorder, insomnia, causes, suggestions, problems, investigation, drowsiness, analysis, reprint, drug dosage, relapse |
| Emotional support | rehabilitation, thanks, farewell, strength, love, help, hope, blessing, victory, happiness, despair, boycott, suicide, pain, bad, happy, death, experience, share, selfregulation, diary, anxiety, pessimism, meaning, fear, memory, life, donation, story, mood, good, get out of depression, get rid of, psychology, perception, give up, darkness, persistence |
